# GaitRec-Net: A Deep Neural Network for Gait Disorder Detection Using Ground Reaction Force

**DOI:** 10.1155/2022/9355015

**Published:** 2022-08-22

**Authors:** Chandrasen Pandey, Diptendu Sinha Roy, Ramesh Chandra Poonia, Ayman Altameem, Soumya Ranjan Nayak, Amit Verma, Abdul Khader Jilani Saudagar

**Affiliations:** ^1^National Institute of Technology, Meghalaya, India; ^2^Department of Computer Science, CHRIST (Deemed to be University), Hosur Road, Bangalore, Karnataka, India; ^3^Department of Computer Science and Engineering, College of Applied Studies and Community Services, King Saud University, Riyadh 11533, Saudi Arabia; ^4^Amity School of Engineering and Technology, Amity University Uttar Pradesh, Noida, India; ^5^Department of Computer Science & Engineering and University Centre for Research & Development, Chandigarh University, Mohali, 140413 Punjab, India; ^6^Information Systems Department, Imam Mohammad Ibn Saud Islamic University (IMSIU), Riyadh 11432, Saudi Arabia

## Abstract

Walking (gait) irregularities and abnormalities are predictors and symptoms of disorder and disability. In the past, elaborate video (camera-based) systems, pressure mats, or a mix of the two has been used in clinical settings to monitor and evaluate gait. This article presents an artificial intelligence-based comprehensive investigation of ground reaction force (GRF) pattern to classify the healthy control and gait disorders using the large-scale ground reaction force. The used dataset comprised GRF measurements from different patients. The article includes machine learning- and deep learning-based models to classify healthy and gait disorder patients using ground reaction force. A deep learning-based architecture GaitRec-Net is proposed for this classification. The classification results were evaluated using various metrics, and each experiment was analysed using a fivefold cross-validation approach. Compared to machine learning classifiers, the proposed deep learning model is found better for feature extraction resulting in high accuracy of classification. As a result, the proposed framework presents a promising step in the direction of automatic categorization of abnormal gait pattern.

## 1. Introduction

One of the most natural and frequent human characteristics is walking. However, it is one of the most complicated occurrences from an analytical perspective. The brain, nerves, and muscles work together to do this. Physiotherapists, orthopedists, and neurologists have long studied human motion in order to assess a patient's condition, rehabilitation, and therapy [1] Gait is the pattern of limb movement during locomotion that contains a variety of information about human individuals. Gait analysis has traditionally been done subjectively through visual assessment, but now, it can be done objectively and effectively due to new technologies. The main aim is to find out the problem that affects the patient's gait pattern [2] As a result, it is frequently utilized in various fields, including affect analysis, sport science, health, and user identification. A variety of sensing modalities, such as wearable sensors connected to the human body, such as accelerometers, gyroscopes, and force and pressure sensors, can be used to record gait data. Nonwearable gait recognition systems are commonly referred to as vision-based gait recognition since they rely heavily on vision. These devices use image sensors to gather gait data from far away distances with no cooperation from the participants. In a medical environment, gait analysis and research can aid in the diagnosis and monitoring of illnesses that affect gait. As a result, automated gait analysis is becoming increasingly common.

Currently, various types of sensors can be used to obtain a rich classification of gait detailed information. Floor sensors are one of the types which can be used to detect GRF measurements or the pressure employed on each area under the foot [3]. The equipment utilized is restricted to constrained areas and provides little information for abnormal gait categorization. The technique utilizing foot pressure data, studied for person identification by various organizations, is a different method than using a camera. GRF quantification is a common method for physicians to impartially specify human locomotion and explain and assess gait patients' detailed performance [4] Force plates are used to calculate the ground reaction forces produced by a body standing on or moving over them. A load cell is an electrochemical device that measures forces on a force plate. Load cells include piezoelectric elements, beam loads, and strain gauge cells. The sensors flex when force is applied to the plate, resulting in measurable voltage changes proportional to the applied force. By orienting the sensors in various directions, the direction and amount of forces in 3D may be determined. It can acquire information such as the center of force, pressure, and the moment around each axis.

Gait, a typical human behavior, can reveal mental illnesses such as depression, dementia, intellectual impairment, and musculoskeletal problems such as joint deformity. [5] An essential aspect used in delivering and estimating the patient's rehabilitation and therapy is a detailed evaluation of the part of activity completed throughout the day. Individuals with lower leg impairments undergo therapy to help them recover motor function in their lower legs. Walking is a pattern-repeating motion that maintains static and dynamic balance. A typical gait sequence begins with one foot tapping the ground and ends with the same foot pounding the ground. A gait cycle may be broken down into stance (when the foot makes contact with the ground) and swing (when the leg does not touch the ground) and then into gait tasks such as ipsilateral, contralateral feet off, and foot contact.

In recent years, some automatic analytic techniques based on artificial intelligence algorithms have been published to help physicians detect and categorize certain gait patterns into clinically significant categories. Neural networks, convolutional neural networks (CNN), Long Short-Term Memory (LSTM), Support Vector Machine (SVM), nearest neighbor classifiers, and other clustering algorithms are among the artificial intelligence methods used in this area. The input data format has a big impact on how well these approaches work. Pataky et al. [6] used dynamic plantar pressure data, picture processing, and feature extraction to achieve high subject recognition accuracy (99.6%). Their discovery highlighted the uniqueness of intersubject pressure patterns, implying that foot pressure-based recognition might have a broad range of applications in the security and health industries. Gul et al. [7] implemented a 3D convolutional neural network architecture for gait identification that employs a comprehensive approach in the form of gait energy images (GEI). This simplified representation captures the shape and motion properties of the human gait. OULP and CASIA-B, two of the biggest openly accessible datasets with significant age and gender variation, were used to test the network. Khokhlova et al. [8] suggested a gait model based on data from the Kinect v.2 sensors; these sensor-generated skeletal sequences are broken down into repeated sections (gait cycles). The low-limb flexion characteristics are then computed for each gait cycle using Kinect orientation data. These characteristics are employed in a machine learning-based gait model.

To become acquainted with the distinction between normal and pathological gait based on kinematic properties sensor, an ensemble LSTM-based architecture is trained using data from a group of people. Lee et al. [9] suggested a deep learning-based gait-type classification technique that uses a smart insole with different sensor clusters to analyses gait data, including a pressure sensor cluster, a gyrosensor cluster, and an acceleration sensor cluster. Using a deep convolutional neural network (DCNN), the feature of the gait pattern was extracted. Experiments demonstrated that the developed technique has a greater than 90% recognition accuracy for seven different gait types (walking, running, stair climbing, hill descending, stair descending, fast walking, and hill-climbing). They were using a machine learning approach; Farah et al. [10] detect gait from thigh kinematics. During walking, 31 able-bodied subjects were measured for thigh angular velocity, knee angle, and thigh acceleration (10 strides each). The characteristics were retrieved using a 0.1-second sliding window after splitting the strides into loading response, swing, terminal swing, and push-off. The knee angle parameter was used to classify gait phases with and without it. The Decision Tree J-48 with knee angle properties was the best classifier, with the second-highest categorization accuracy of 97.5 per cent and the least MAE (mean absolute error) of 0.014. Based on deep learning evaluation of sagittal knee-joint angle obtained by one electrogoniometer, Di Nardo et al. [11] propose a novel method for binary gait phase categorization and gait event prediction. Neural networks were used by Horsak et al. [12] to separate imitate gait (e.g., leg length disparity) using features from lower-leg joint-angle data. In contrast, Manap et al. [13] used force platform recordings of foot-ground reaction forces to classify normal and diseased gait. The dataset used a pressure-based sensor for walking, and it is classified using kernel-based principal component analysis (KPCA) and SVM in [14]. However, when data is projected on orthogonal axes while conserving the most variance, this approach reflects fluctuations that are not useful for walking classification. As a result, it is inappropriate to categorize data with several classes or variations in gait patterns. All over, the above study suggest a need of highly effective automated method for gait abnormality detection with effective results for early detection of abnormality in real time.

The main contribution of this paper is use of artificial intelligence techniques for the classification of healthy and pathological gait conditions of the patient based on ground reaction force. The experimental outcomes show that the proposed CNN architecture gives the highest average accuracy of 91.62%. The proposed framework presents a promising step in the direction of automatic categorization of abnormal gait. Precision, F1 score, and sensitivity are performance evaluation metrics used to evaluate the machine learning classifiers and proposed deep learning architecture GaitRec-Net.

The key characteristics of the study are as follows:

1. Artificial intelligence technology is used to classify healthy and pathological conditions of the patient based on ground reaction force
2. A deep neural architectures 1-D GaitRec-Net architecture is proposed. A machine learning classifier, SVM, KNN, and Naïve Bayes, using 5-fold cross-validation is used for binary classification (gait disorder and healthy control).
3. An optimized layer, batch normalization, and dropout have been chosen for the suggested architecture to minimize misclassification and overfitting issues
4. The performance of the proposed model GaitRec-Net CNN showed the highest accuracy of 91.624% compared to other machine learning classifiers

The rest of this paper is structured as follows. The data used in the paper is discussed in Section 2. The proposed methodology is described in Section 3. The experiments and results are covered in Section 4, while Section 5 wraps up the paper and offers recommendations for future research reference.

## 2. Materials and Methodology

The proposed system is aimed at classifying the gait disorder and healthy subjects from pressure-based data.

Data were obtained from both the healthy control and the gait disorder patients after clinical and histopathological evaluations. GaitRec is a preprocessed dataset that contains 75,732 bilateral trials. Before feeding the dataset, the preprocessing such as data annotation, denoising, data normalization, and substituting the NAN values with mean is done. The dataset is divided into training and testing. The 5-fold cross-validation is applied to evaluation by machine learning (SVM, KNN, and Naïve Bayes) and the proposed (GaitRec-Net) deep learning architecture.

**Figure 1:**
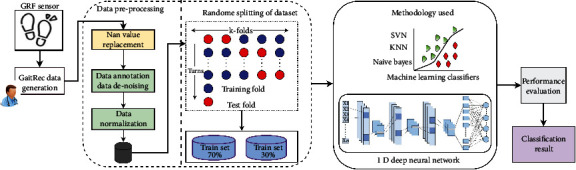
Block diagram of the proposed system.

The proposed system's schematic diagram is presented in Figure 1. It is split into 3 parts for the automatic classification of gait disorder. The first section is the data collection and preprocessing, in which the GaitRec dataset is used after proper preprocessing such as data annotation, data denoising, and data normalization. The NAN value is substituted, and the salient gait features are extracted, and statistically based feature selection is performed using the extracted features. The normalization and ranking procedures are then applied to these selected salient gait features, followed by a classification algorithm.

The second module is about training, and it employs an *n*-fold cross-validation (CV). At random, the total samples were split into *n* equal-sized subgroups. The rest of the *n* − 1 subset was used as a training set, with only one subset kept as a test set for model validation. The cross-validation technique was then repeated *n* times. As a test set, each subset was utilized exactly once because *n* was traditionally equal to the number of courses; here, 5-fold CV is performed for a thorough examination of the machine learning classifier and the proposed GaitRec-Net architecture. Data is divided into two sections for each fold: training and testing, with the classifiers being trained and tested for each fold. The final categorization outcome is calculated as a probability value of having a gait problem or not, in the third module using the machine learning classifier and proposed GaitRec-Net architecture. The proposed GaitRec-Net architecture was compared to several traditional machine learning techniques, which are widely utilized to address classification issues in the intersubject implementation. The remaining subsections go over the entire process in great depth.

## 3. Dataset Description

The data considered in this study are from the currently available medical gait database and a unique pathological dataset called GaitRec [12], which is kept by an Austrian Workers' Compensation Board rehabilitation center (AUVA). The data was collected between 2007 and 2018 while in clinical practice. A physical therapist gathered GRF readings from 2085 patients with gait disorders (calcaneus, ankle, knee, and hip) and samples from 161 healthy (*N*) based on each patient's known medical diagnosis, including men and women of varying physical features and gender. Patients having ligament ruptures, joint replacement surgery, fractures, and other associated diseases fall under the gait disorder (GD) categories. One or more measurement sessions were completed by each patient. Each session consisted of 8 recordings of two continuous steps. In this study, each bilateral recording is known as a trial. As a result, the used dataset includes 75,723 bilateral trials, as shown in Table 1.

### 3.1. Data Collection and Preprocessing

**Figure 2:**
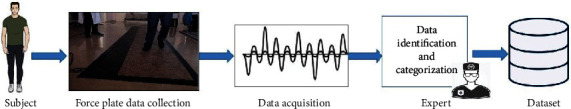
Schematic of gait data collection.

Gait assessment was conducted on a 10-meter pathway with 2 force plates (Kistler, Type 9281B12) implanted in the middle. The force plates were arranged in a row so that an individual could walk over them by putting one foot on each plate. Subjects were asked to walk without an assistive technology at a self-choose walking speed on a 10-meter walkway with 2 force plates inserted in the middle to measure bilateral GRF (ground reaction force, which occurs when the weight of the body acts vertically downward on the ground).  Figure 2 shows the data collection and labeling process of the gait dataset.

People walked at 3 distinct rates (mean and variance, m/s). Using a 2000 Hz sampling rate, the 3 analogue ground reaction force signals (anterior-posterior, vertical, mediolateral, and force elements) and the center of pressure (COP) were transformed into digital data. In the force plate coordinate system, COP and GRF were acquired. Raw feeds were only accessible downsampled to 250 Hz according to the center's internal standards. Prevent signal peaks and noise at beginning and end of the signals, and all force sample was transferred to a 25 N threshold, following which the COP was computed. To minimize errors in COP computation at low force levels, the COP coordinates for the postprocessed dataset were anterior-posterior, and mean-centered coordinates were zero-centered. The processed signals were then time-normalized to 100 per cent stance and filtered with a second-order low-pass Butterworth filter with 20 Hz cut-off frequency to minimize the noise. The amplitude values of the three force components, namely, anterior-posterior (AP), mediolateral (ML), and vertical (V), were expressed as a multiple of body mass by dividing the force by the product of body mass times acceleration due to gravity. The foot length determined throughout each session, calculated as a product of foot length, was used to normalize the COP waveforms from each trial.

In terms of annotation, a well-experienced physical therapist manually annotated the dataset based on each patient's accessible medical diagnosis. The annotation labels are made up of two strings joined by an underscore “X_xxx,” where “*X*” stands for the general anatomical joint level where the orthopedic impairment occurred. The second string (“xxx”) is joint-dependent and provides a more thorough localization. The classification in this research is based on postprocessed data. The precision of the plates was not particularly verified during the data-gathering period. The force plates and measuring equipment, on the other hand, were examined and maintained regularly throughout clinical practice. The forces that were employed in the dataset are described in Table 2.

### 3.2. Classification Algorithm

In this subsection, four supervised algorithms: Naive Bayes, KNN, SVM, and a proposed GaitRec architecture, are used for classification purpose, where a set of the feature vector is given as an input for training data and produces an inferred function, which can be used for validating the test data. Here, in Naïve Bayes, normal distribution is estimated for each class by computing the mean and standard deviation of the training data, a Gaussian distribution (var_smoothing = 1*e* − 09) is widely used to create the probabilistic model for biomechanical gait data. The data is then assigned to the most likely class using a decision rule [13]. Create a training database, *t*, and assign it a class label, *L*. Following that, with*L*classes labeled as N1, N2,....., NL, each sample will be defined by an*n*-dimensional vector,*Y* = Y1, Y2, ⋯Y*n*, where*n*represents the measured characteristics,X1, X2, ⋯X*n*, respectively, fitting the 1D training data results in the creation of a Naïve Bayes classifier object. Following then, a new set will be assigned as the classifier's testing data. Each class testing dataset's posterior probability will be computed; based on the probability, the data is classified between healthy and gait disorder.

In general, SVM is one of the most used machine learning approaches used for gait classification. This is a feature-based classifier that maximizes the margin between distinct classes to create hyperplane borders and is based on Vapnik's statistical learning theory [14]; they treat the learning problem as a quadratic optimization problem with a global optimum and no local minima on the error surface. In this experiment, linear kernel is used which plots a single data item in an *M*-dimensional space (*M*—number of attributes), with the value of each attribute being the value of a particular coordinate. It can be done by finding the hyperplane that distinguishes the two classes. The set of parameters used for SVM is kernel = linear, *C* = 1.0, class_weight = none, and penalty = ^“^l2^”^.

The third classifier is *K*-nearest neighbours (KNN) which is a nonparametric technique categorized by the majority vote of its neighbours. The object is classified as the most general class among its *K*-nearest neighbours. The KNN classifier is a frequently used machine learning method that is one of the simplest. It decides whether to allocate a new point in the feature space to a certain class based on the similarity measure of the distance between the analysed point and the *K*-nearest neighbours (KNN). An object's distance from its neighbours is used to classify it, with the object being allocated to the most common class among its *K*-nearest neighbours. Each item in a multidimensional feature space is represented by position vectors. Euclidean distance is utilized to calculate the distances between training and test vectors. The set of parameters used for KNN is neighbours = 3, weight = uniform, metrics = ^“^Minkowski^”^, and *p* = 2.

### 3.3. Proposed GaitRec-Net Architecture

**Figure 3:**
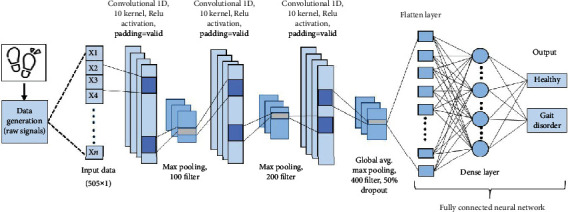
Proposed GatiRec-Net architecture.

1D CNN is widely used for feature extraction and signal data categorization because of the advancement of computer power and the huge volume of labeled data. 1D CNNs are feed-forward networks in which information passes from the input to the output in only one way. In a 1D CNN, the input, usually a one-dimensional tensor, is processed layer by layer. Convolutional neural networks are advantageous for sequence classification because they can learn directly from raw time series data, reducing the requirement for domain knowledge to manually generate input features. The model should learn an embedding layer from the signal data and, in theory, perform similarly to models trained on a dataset that has been artificially augmented. The kernel slides overall spatial places in the input “signal” when the convolution layer is used.  Figure 3 represents the layers of the proposed architecture.

An input layer in the proposed 1D-GaitRec-Net architecture has a dimension of 505∗1, and an output layer has a dimension of 2. After multiple experiments, the metrics (stride, filters, and kernel) were optimal. The network is set up so that it learns from small, localised patterns in the gait signal. Midlevel features are created by combining these tiny and localised patterns. More sophisticated and high-level characteristics are created by combining these midlevel features. These characteristics are employed in the gait phase detection classification job.

The suggested model's filter convolves the input “signals” by moving one unit at a time with padding. Numerous response maps can be obtained by using multiple convolution layers. In contrast to the previous machine learning manual procedures, the convolution operation is considered the foundation of a CNN architecture, where feature extraction occurs automatically, saving time throughout the process. In this model, there are three convolution layers, 2 maxpooling layers, 1 average pooling layer, 1 dropout layer, and 1 dense layer, as mentioned in Table 3. Here, every convolutional layer with 10∗10 kernel size and 100∗200 filter size is followed by a maxpooling layer having activation function ReLu, to capture the nonlinear relationship of the features. The size of the input is not changed by a ReLu layer. Rectified Linear Unit (ReLu) activation means*A*and*B*have the same size, as represented mathematically in equation ((1)).The ReLu activation function is used in all of the layers until the last one.

(1) $$\begin{array}{c} B\left(N\right)=\max\left(0,A\left(N\right)\right). \end{array}$$

The softmax activation function is used in the dense layer to normalize the outputs into probabilities of the two classifications. The softmax function is based on Luce's choice axiom [15] and is represented mathematically in the following equation:

(2) $$\begin{array}{c} Y{\left(\overset{⟶}{a}\right)}_{i}=\frac{e^{a_{i}}}{\sum_{j=1}^{k}\;e^{a_{j}}}. \end{array}$$

The weights are updated using the Adam optimizer. Following a convolution layer with ReLu activation, use a flatten layer to eliminate all but one dimension. The dropout size is 0.5, and it is applied as the last layer to reduce overfitting. Finally, a dense layer of two neurons depicts two types of people: healthy and gait disorder. TensorFlow [16] and Keras [17] APIs in Python 3 were used to create the above architecture.

### 3.4. Hyperparameter Optimisation

The GaitRec-Net architecture uses 5-fold cross-validation with suitable hyperparameter tuning. While training in every fold, the overfitting of the data is avoided using early stopping conditions with 50 epochs. The hyperparameters utilized to train the developed GaitRec-Net architecture for the categorization of healthy and gait disorders are listed in Table 4.

Various parameters such as the batch size, padding, optimizer, epoch, and loss based on early stop criteria have been calculated experimentally through a series of experiments. The binary crossentropy is being used together with Adam (Adaptive Moment Estimation) optimization function for loss calculation.

## 4. Experimental Results

The recently Gait-Rec dataset was split into a binary classification for training three machine learning classifiers and a proposed CNN architecture. The presented GaitRec-Net architecture was trained on training data and validated on test data using fine-tuned parameters. The trained model can predict whether the patient has a gait disorder or not. The training is done on a workstation with twin Intel Xeon Platinum 8168, 2.7 GHz, 24-cores, and 64 GB RAM running 64-bit Windows 10 Pro. The graphics card is a 16X NVIDIA® Tesla® V100 with 512 GB of total graphics memory. In the Jupyter Notebook environment, the programming language utilized is Python version 3.6.9.

The various machine learning and proposed GaitRec-Net approaches for gait disorder classification are analysed using 5-fold cross-validation based on various performance metrics: precision, accuracy, recall or sensitivity, and F1 score. These performance matrices are obtained using true positive (TP), false negative (FN), false positive (FP), and true negative (TN), which can be calculated using the obtained confusion matrices as shown in Table 5. To make the GaitRec-Net trained model more resilient, an exhaustive analysis is done utilizing the model's performance matrix. In equations (3) to (6), the matrix components are specified on which the model's performance is assessed.

(3) $$\begin{array}{c} \mathrm{Pr}=\frac{\left(\text{TP}\right)}{\left(\text{TP}+\text{FP}\right)}\times 100\%, \end{array}$$

(4) Re=TPTP+FN×100%,

(5) F1score=2×Pr×RePr+Re×100%,

(6) $$\begin{array}{c} \text{Ac}=\frac{\text{TP}+\text{TN}}{\text{Total number of gait signal}}\times 100\%, \end{array}$$

where TP (true positive) denotes the samples that are associated with the class of healthy condition that are correctly classified as belonging to it, FP (false positive) represents the samples that are associated with gait disorder and are incorrectly classified as belonging to the class of healthy condition. TN (true negative) represents the samples which are actually associated with the class healthy condition and are correctly classified as belonging to gait disorder. FN (false negative) represents the samples that are actually associated with the class of gait disorder but incorrectly classified as belonging to healthy condition. Here, in the above-mentioned equations; Pr denotes precision; Re denotes sensitivity; F1 denotes the F1 score, which is the HM (harmonic mean) of sensitivity and precision; and Ac denotes accuracy, which is the proportion of TP + TN and a total number of gait signals.

**Figure 4:**
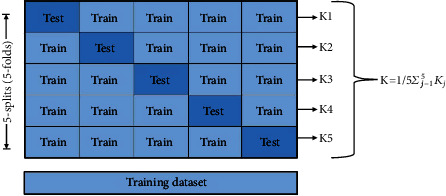
Fivefold cross-validation process.

In the initial step, the various machine learning techniques and GaitRec architecture were trained and tested for classification. For an extensive evaluation of the classifiers, data from healthy control and gait disorder were fed to several algorithms using the 5-fold cross-validation approach. The artificial intelligence algorithm performance cannot be explained purely by splitting the dataset into separate training and testing sets. Cross-validation is distinguished by the fact that it employs all of the data points in the dataset, resulting in minimal bias. Dataset was split into 5-fold cross-validation, where onefold is used for testing purposes, and another fourfold is used for training purposes. During fivefolds, there was no data sample collision in the testing set.  Figure 4 represents the process of fivefold cross-validation.

In this paper, the dataset is tested and trained using the proposed GaitRec-Net architecture and various machine learning classifiers using 5-fold cross-validation. The data were separated into five equal sections, where 70% is for the training set, and the remaining 30% is used to test the model's effectiveness. For the performance test, the confusion matrix and various performance measures such as precision, F1 score, and recall are used. Each set's training and testing results (loss and accuracy) are evaluated. To determine its worth, the outcome was also compared to existing methodologies.

The different output parameters of the proposed GaitRec-Net model in the GaitRec dataset are explained in Table 6. Other variables are used in a 5-fold cross-validation (validation accuracy, validation loss, training accuracy, and training loss) on that fold (epoch, validation samples, and training samples). The Adam function is employed for optimization, with a learning rate of 0.001. The suggested model architecture has a total of 1,002,502 trainable parameters. For each fold, the model is trained using 100 epochs and a batch size of 128. For the GaitRec dataset, the achieved average validation and training accuracies are 91.622% and 91.624%, respectively, and the obtained average validation loss and training loss are 0.23186 and 0.24582, respectively. The suggested system correctly classified gait signals as healthy and gait disorder with an average accuracy of 91.624%.

For both sets of training, Table 5 compares the ground-truth labels to the confusion matrix for all 5-fold binary classification predictions (healthy condition and gait disorder). In a 1D CNN, more training data leads to higher accuracy; thus, more accuracy with a larger dataset is reflected in the confusion matrix. In the performed experiment, fifth-fold 99.11% samples are correctly classified as healthy condition and 74.61% samples are correctly classified for gait disorder in comparison with other folds.

Table 7 represents different performance matrices such as sensitivity, precision, and F1 score of the proposed deep learning architecture (GaitRec-Net) that are used for the classification of gait signals on the GaitRec free-access dataset for all 5-fold cross-validation, where the highest precision of healthy control is 77% at fold five, the highest recall is 33% at fold one, and the highest F1 score is 44% at fold four. For gait disorder, the highest precision is 93% at fold one, three, and four; the highest recall is 99% on each fold; and the highest F1 score is 96% at fold one, four, and five. Average accuracy of each classifier with respect to each fold is presented in Table 8.

Machine learning classifiers such as SVM, Naïve Bayes, and KNN are also trained on the same data configuration with 70% for training and the remaining 30% to test the model. Fivefold CV is applied to these classifiers. The various accuracies at each fold are presented in Table 8. The proposed deep learning model achieved the highest average accuracy of 91.624% compared to other machine learning classifiers.

**Figure 5:**
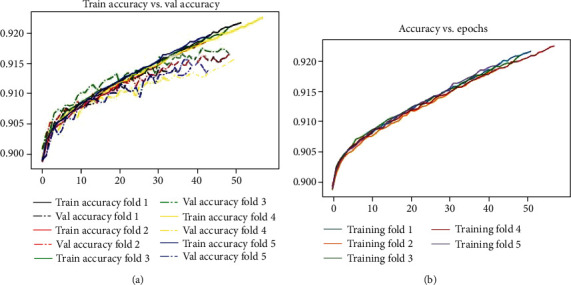
The plot of (a) training accuracy vs. validation accuracy in each fold. (b) Accuracy vs. epoch in each fold.

Figure 5(a) shows the graph of training and validation accuracy on each fold of 5-fold CV in the given GaitRec dataset. The solid lines show the training accuracy, and the dotted lines show the validation accuracy.  Figure 5(b) shows the graph of training accuracy on each epoch of GaitRec-Net architecture at each 5-fold cross-validation.

Table 9 shows the comparison table of previous work and the proposed work for binary classification of gait disorder using pressure-based data. Fricke et al. [18] reported higher accuracy of 91.9% on a CNN with 37 subjects. The proposed method achieved 91.624% accuracy for test data. This is a significant performance improvement, indicating the GaitRec-Net network's supremacy on gait data.

The performance of various machine learning techniques and the proposed CNN architecture was analysed, and the best-performing models were chosen as the final approach. To avoid underfitting, ensure that the learning algorithm or method fits the data well enough and achieves accurate results. In order to avoid overfitting, resampling approaches were used in this work to evaluate method accuracy while analysing machine learning techniques. The most frequently used resampling approach is *n*-fold cross-validation, which is the preferred method in learning algorithms for testing model accuracy in unrevealed data. It trains and tests the *k*-times model on various subsets of training data to give an assessment of a model's performance using unseen data. The proposed deep learning-based technique on 5-fold cross-validation yielded very accurate generalised results for the trained models.

## 5. Conclusion

In this research, an automatic deep learning framework was proposed which classifies healthy and gait disorder. The proposed GaitRec-Net is based on 1-dimensional convolutional neural network architecture. The experiment involves three machine learning classifiers and one proposed deep learning architecture. The overall average classification accuracy of the proposed architecture was 91.624% using 5-fold cross-validation. The following are the three machine learning algorithms, i.e., SVM (89.998%), Naïve Bayes (55.244%), and KNN (91.296%). The work reveals that the proposed deep learning-based architecture may be used to build noninvasive tools for the classification of gait in clinical settings. Future research will concentrate on broadening the scope of the study by including additional diseased populations and improving classification accuracy and multiclass gait impairment classification.

## Figures and Tables

**Table 1:** Represent a total number of data in the GaitRec dataset.

Class		Subjects	Sex		Body mass (kg)	Mean	Bilateral trials
			Male	Female			
Healthy (*N*)		211	104	107	73.9	15.6	7,755
Gait disorder (GD)	Hip	450	373	77	82.4	15.6	12748
	Knee	625	426	199	84.3	18.6	19873
	Ankle	627	498	129	87.0	18.0	21386
	Calcaneus	382	339	43	84.0	14.5	13970
Total		2295	1740	555	83.6	17.3	75,732

**Table 2:** Description of postprocessed GaitRec dataset.

Variables	Description
Vertical GRF	Represent the postprocessed GRF
Anterior-posterior GRF	Represent the breaking and propulsive shear forces after they have been postprocessed
Mediolateral GRF	Mediolateral shear force after postprocessing
COP anterior-posterior	COP coordinate in walking direction after postprocessing
COP mediolateral	COP that has been postprocessed in mediolateral direction coordinates

**Table 3:** A proposed GaitRec-Net model summary with various parameters.

S. No.	Layer (type)	Output shape	Kernel size	Activation function	Parameters
0	Input layer	(None, 505, 1)			
1	Conv1D	(None, 496, 100)	10	ReLu	1100
2	MaxPooling1D	(None, 248, 100)	—	—	0
3	Conv1D	(None, 48, 200)	10	ReLu	200200
4	MaxPooling1D	(None, 24, 200)	—	—	0
5	Conv1D	(None, 3, 400)	10	ReLu	800400
6	Global_average_pooling1D	(None, 400)	—	—	0
7	Dropout	(None, 400)	—	—	0
8	Dense	(None, 2)		Softmax	802
	Nontrainable parameters				0
	Trainable parameters				1,002,502
	Total parameters				1,002,502

**Table 4:** Hyperparameters and their values.

Hyperparameter	Value
Batch size	128
Epochs	100
Multiprocessing	“False”
Padding	“Valid”
Optimisation	“Adam”
Early stopping	50 epochs
Loss	“Binary crossentropy”

**Table 5:** The confusion matrix for the proposed GaitRec-Net model on the GaitRec dataset.

		1-fold		2-fold		3-fold		4-fold		5-fold	
Ground truth	HC	98.71%	1.28%	98.63%	1.37%	98.54%	1.45%	98.65%	1.34%	99.11%	00.88%
	GD	70.10%	29.10%	70.38%	29.61%	69.72%	30.27%	68.82%	31.17%	74.61%	25.39%
		HC	GD	HC	GD	HC	GD	HC	GD	HC	GD
		Prediction		Prediction		Prediction		Prediction		Prediction	

**Table 6:** Fivefold cross-validation result of GaitRec-Net architecture on the GaitRec dataset.

Fold	Training samples	Valid samples	Loss	Ac.	Valid loss	Valid Ac.	Total epochs
1	271992	30936	0.2299	0.9169	0.2999	0.9168	52
2	271960	30968	0.2352	0.9157	0.2351	0.9157	43
3	271862	31066	0.2335	0.9154	0.2334	0.9154	49
4	271774	31154	0.2299	0.9171	0.2299	0.9171	58
5	272093	30835	0.2308	0.9161	0.2308	0.9161	44
		Average:	0.23186	0.91624	0.24582	0.91622	49.2

**Table 7:** Fivefold cross-validation result on the GaitRec dataset.

Fold	HC			GD		
	Pr	Re	F1	Pr	Re	F1
1	0.73	0.33	0.42	0.93	0.99	0.96
2	0.71	0.30	0.42	0.92	0.99	0.95
3	0.70	0.30	0.42	0.93	0.99	0.95
4	0.73	0.31	0.44	0.93	0.99	0.96
5	0.77	0.25	0.38	0.92	0.99	0.96

**Table 8:** The average accuracy of classifiers at each fold.

SN	Classifier	Onefold	Twofold	Threefold	Fourfold	Fivefold	Accuracy (%)
1	SVM	90.01	90.14	89.98	89.88	89.98	89.998
2	NB	55.29	55.16	55.24	55.33	55.20	55.244
3	KNN	91.28	91.24	91.34	91.32	91.30	91.296
4	GaitRec-Net	91.68	91.57	91.54	91.71	91.61	91.622

**Table 9:** State of the art of previous work.

Reference	Dataset	Methodology	No. of subjects	Classification & accuracy
[19]	Private dataset	Logistic regression; SVM & MARS	8	Binary class
				MARS = 88.3%; logistic regression = 68.5% & SVM = 84.8%
[20]	MFC data	SVM	58	83.3%
[18]	Private dataset	PCA + (SVM, KNN) & CNN	37	Binary class
				CNN = 91.9%; SVM = 67.6% & KNN = 48.7%
				Multiclass
				CNN = 83.8%; SVM = 51.4% & KNN =32.4%
[21]	Private dataset	PCA + linear SVM; RBF SVM	440	Binary class
				Linear SVM = 90.8%; RBF SVM = 89.1%
				Multiclass
				Linear SVM = 54.3; RBF SVM = 51.2%
[22]	Private dataset	KPCA + (SVM; ANN; random forest[RF])	239	Multiclass
				SVM = 89%; ANN = 90% & RF = 73%
Proposed method	GaitRec dataset	SVM; KNN; Naïve Bayes; 1D CNN	2295	Binary class
				SVM = 89.998%; KNN = 91.296%; Naive Bayes = 55.244% & 1D CNN = 91.624%

## Data Availability

The data presented in this study are available on request from the corresponding author.
